# Tissue Advanced Glycation End Product Accumulation and Its Association with Clinical and Laboratory Features, Inflammatory Indices, and Comorbidities in Rheumatoid Arthritis and Ankylosing Spondylitis: A Case–Control Study

**DOI:** 10.3390/ijms27094027

**Published:** 2026-04-30

**Authors:** Altuğ Güner, Taner Dandinoğlu

**Affiliations:** 1 Department of Rheumatology, Bursa City Hospital, 16110 Bursa, Türkiye; 2 Department of Physical Medicine and Rehabilitation, Bursa City Hospital, 16110 Bursa, Türkiye; dandinoglu@gmail.com

**Keywords:** advanced glycation end products, skin autofluorescence, rheumatoid arthritis, ankylosing spondylitis, inflammation, disease activity, comorbidity

## Abstract

Advanced glycation end products (AGEs) accumulate under chronic inflammation and metabolic stress and may contribute to long-term tissue damage. Skin autofluorescence (SAF) enables non-invasive assessment of tissue AGE accumulation, but data in inflammatory rheumatic diseases remain limited. The present study evaluated AGE-SAF levels in patients with seropositive rheumatoid arthritis (RA) and ankylosing spondylitis (AS) and investigated their associations with disease activity, inflammatory markers, metabolic parameters, and comorbidities. Patients with RA and AS, along with healthy controls, were included. AGE-SAF was measured non-invasively. Disease activity was assessed using the Disease Activity Score in 28 joints (DAS28) and the Bath Ankylosing Spondylitis Disease Activity Index (BASDAI). Between-group comparisons were performed both crudely and after multivariable adjustment for age, sex, and body mass index (BMI). Logistic regression and receiver operating characteristic (ROC) analyses were used to describe the exploratory discriminative performance of AGE-SAF, and propensity score analyses were conducted as sensitivity analyses to address baseline imbalance. In crude comparisons, AGE-SAF levels were higher in RA than in AS and controls, and higher in AS than in controls (*p* < 0.001). After adjustment for age, sex, and BMI, AGE-SAF remained significantly elevated in both RA (β = 0.440, 95% CI 0.298–0.583, *p* < 0.001) and AS (β = 0.304, 95% CI 0.183–0.425, *p* < 0.001) compared with controls; however, the difference between RA and AS was no longer statistically significant (β = 0.136, 95% CI −0.051 to 0.323, *p* = 0.154). Exploratory ROC analyses showed good discrimination for RA versus controls (AUC = 0.851) and moderate discrimination for AS versus controls (AUC = 0.695), whereas discrimination between RA and AS was limited (AUC = 0.670). In overlap-weighted sensitivity analysis, the RA-AS difference remained non-significant (β = 0.161, *p* = 0.293). AGE-SAF is elevated in inflammatory rheumatic diseases compared with healthy controls, and this elevation persists after adjustment for age, sex, and BMI. Although crude AGE-SAF values were higher in RA than in AS, this difference attenuated after confounder adjustment, indicating that a substantial part of the between-disease difference is attributable to demographic and treatment-related imbalance. AGE-SAF may therefore reflect cumulative disease-related and vascular–metabolic burden across both diseases rather than a disease-specific phenomenon.

## 1. Introduction

Rheumatoid arthritis (RA) and ankylosing spondylitis (AS) are chronic inflammatory rheumatic diseases characterized by persistent immune activation, systemic inflammation, and progressive structural damage, with distinct pathophysiological mechanisms but overlapping inflammatory and cardiometabolic consequences. Beyond musculoskeletal involvement, these conditions are associated with metabolic alterations and an increased burden of comorbidities, particularly cardiovascular disease (CVD). Chronic inflammation and oxidative stress are central to endothelial dysfunction and accelerated atherosclerosis observed in patients with inflammatory rheumatic diseases [1,2].

From a mechanistic perspective, advanced glycation end products (AGEs) may provide a critical link between chronic inflammation, metabolic dysregulation, and long-term tissue damage [3,4,5]. AGEs are formed through non-enzymatic glycation and oxidation of proteins and lipids under conditions of oxidative stress and hyperglycemia. Chronic inflammation may further accelerate AGE formation by increasing reactive oxygen species and enhancing glycoxidation pathways [5,6,7]. Once formed, AGEs interact with the receptor for advanced glycation end products (RAGE), leading to activation of intracellular signaling pathways, particularly nuclear factor kappa B (NF-κB), which may in turn promote the production of pro-inflammatory cytokines such as interleukin-1 (IL-1), interleukin-6 (IL-6), and tumor necrosis factor alpha (TNF-α). This AGE-RAGE-NF-κB axis may contribute to a self-perpetuating cycle of inflammation and oxidative stress that may ultimately contribute to endothelial dysfunction, vascular injury, and progressive tissue damage [4,6,8,9,10,11].

Growing evidence suggests that AGE accumulation may contribute to the pathophysiology of rheumatic diseases. Elevated AGE-SAF levels have been reported in patients with RA and are associated with systemic inflammation, endothelial dysfunction, and increased cardiovascular risk [12]. These findings indicate that AGE-SAF may reflect cumulative inflammatory and metabolic stress in chronic inflammatory conditions.

AGE-SAF levels may also be influenced by chronic comorbid conditions beyond inflammatory rheumatic disease itself. In particular, diabetes mellitus, chronic kidney disease, and cardiovascular disease have all been associated with increased tissue AGE burden and higher skin autofluorescence in previous studies [13]. This broader clinical context is important when interpreting AGE-SAF values, because tissue AGE accumulation may reflect the combined contribution of rheumatic disease and coexisting metabolic or vascular comorbidity rather than a single disease process.

Tissue AGE accumulation can be assessed non-invasively using AGE-SAF, which has emerged as a reliable surrogate marker of long-term AGE deposition. AGE-SAF measurement is rapid, painless, and reproducible, and has been widely used to evaluate cumulative glycation stress in metabolic and cardiovascular diseases. Several studies have demonstrated that AGE-SAF correlates with vascular complications and cardiometabolic risk, supporting its potential role as a biomarker of systemic disease burden [14,15]. However, no universally validated AGE-SAF cut-off for susceptibility to rheumatic disease has been established.

In recent years, composite inflammatory indices derived from routine laboratory parameters have gained increasing attention as markers of systemic inflammation and disease activity. Indices such as the systemic immune-inflammation index (SII), the hemoglobin–albumin–lymphocyte–platelet (HALP) score, and the C-reactive protein–albumin–lymphocyte (CALLY) index integrate hematological and biochemical components reflecting both inflammatory and nutritional status. These indices have been increasingly investigated as potential indicators of disease activity and prognosis across a range of inflammatory and malignant conditions [16,17,18].

Despite growing interest in AGE biology, the relationship between AGE-SAF and inflammatory indices, laboratory parameters, and comorbidity burden in chronic rheumatic diseases remains insufficiently explored. RA and AS were specifically compared because both are chronic inflammatory rheumatic diseases, yet they differ in clinical phenotype, inflammatory pattern, and comorbidity profile. We hypothesized that AGE-SAF would be elevated in both diseases compared with healthy controls and explored whether any residual between-disease difference persisted after adjustment for demographic and treatment-related factors.

Therefore, the present study aimed to evaluate tissue AGE accumulation assessed by AGE-SAF in patients with RA and AS and to investigate its association with clinical characteristics, laboratory parameters, inflammatory indices, and comorbidities.

## 2. Results

### 2.1. Demographic Characteristics

There was a significant difference in age among the seropositive RA, AS, and control groups (*p* < 0.001). According to pairwise comparisons, the seropositive RA group was significantly older than both the control group and the AS group (adj. *p* < 0.001 for both comparisons). No significant difference in age was observed between the control group and the AS group (adj. *p* = 1.000). The distribution of sex differed significantly among the disease groups (*p* = 0.001). In the seropositive RA group, the proportion of female patients (71.3%) was higher than that of male patients (28.7%). In contrast, in the AS group, the proportion of male patients (57.8%) was higher than that of female patients (42.2%). In the control group, the proportions of male (49.0%) and female (51.0%) participants were similar. Overall, female patients were more prevalent in the seropositive RA group, whereas male patients were more predominant in the AS group. There was also a significant difference in BMI among the groups (*p* = 0.001). In pairwise comparisons, the BMI value of the seropositive RA group was significantly higher than that of the control group (adj. *p* = 0.001). However, no significant difference was found between the seropositive RA and AS groups (adj. *p* = 0.140) or between the AS and control groups (adj. *p* = 0.469) (Table 1). Because age, sex, and BMI differed significantly across groups, confounder-adjusted comparisons of AGE-SAF are presented below.

### 2.2. Clinical Characteristics

Seropositive RA and AS patients were compared in terms of clinical characteristics. The mean disease duration was 6.96 ± 5.51 years in the seropositive RA group and 5.80 ± 5.21 years in the AS group, and no significant difference was observed between the groups (*p* = 0.147). BASDAI was evaluated exclusively in the AS group, with a mean value of 3.42 ± 2.25. The proportions of patients classified as having active disease according to disease-specific activity indices were similar between the groups (*p* = 0.915). In the seropositive RA group, 56.3% of patients were inactive and 43.8% were active, whereas in the AS group these rates were 55.4% and 44.6%, respectively. There was a significant difference in medication use between the groups (*p* < 0.001). In the seropositive RA group, most patients were receiving conventional therapy (77.5%), whereas anti-TNF therapy was more frequently used in the AS group (56.6%). NSAID use was observed only in the AS group (26.5%), while treatments such as tocilizumab and rituximab were used only in the seropositive RA group. Steroid use was significantly higher in the seropositive RA group (*p* < 0.001), with all patients receiving corticosteroids (100%), compared with only 7.2% in the AS group. Similarly, the distribution of steroid doses differed significantly between the groups (*p* < 0.001), with the majority of seropositive RA patients receiving a daily dose of 5 mg. Major organ involvement differed significantly between the seropositive RA and AS groups (*p* = 0.013). Major organ involvement was observed in 7.5% of seropositive RA patients, whereas no cases were detected among AS patients. Among AS patients, HLA-B27 positivity was detected in 72.0% of cases. Regarding the type of involvement, axial involvement was present in the majority of patients (93.9%), whereas peripheral involvement was observed in 6.1%. Additionally, a history of tuberculosis was identified in a small proportion of AS patients (16.7%). The presence of comorbidities was significantly higher in seropositive RA patients compared with AS patients (76.3% vs. 48.2%, *p* < 0.001). When comorbidities were analyzed individually, diabetes mellitus (*p* = 0.029), hypertension (*p* < 0.001), and hyperlipidemia (*p* = 0.013) were more frequent in the seropositive RA group. In contrast, no significant differences were found between the groups regarding coronary artery disease (*p* = 0.154), thyroid disease (*p* = 1.000), chronic kidney disease (*p* = 0.491), or other comorbidities (*p* = 0.854). Osteoporosis was significantly more common in the seropositive RA group (23.8% vs. 3.6%, *p* < 0.001). However, no statistically significant differences were observed between the groups in terms of atherosclerosis (*p* = 0.084) or cerebrovascular disease (*p* = 0.163). Overall, steroid use, the presence of comorbidities, and several cardiometabolic comorbidities were more common in patients with seropositive RA, whereas biological therapy use was more prevalent among AS patients (Table 2).

### 2.3. Laboratory Characteristics

C-reactive protein (CRP) (*p* = 0.032) and erythrocyte sedimentation rate (ESR) (*p* = 0.002) levels were higher in the seropositive RA group, whereas albumin levels were lower (*p* = 0.006). The lymphocyte count was higher in the AS group (*p* < 0.001), while hemoglobin levels were lower in the seropositive RA group (*p* < 0.001). Creatinine levels were higher in the AS group (*p* = 0.041). When lipid and metabolic parameters were examined, high-density lipoprotein (HDL) levels were higher in the seropositive RA group (*p* < 0.001), and total cholesterol levels were also higher in the seropositive RA group (*p* = 0.001). Hemoglobin A1c (HbA1c) (*p* = 0.024) and vitamin B12 levels (*p* = 0.039) were also higher in the seropositive RA group. The difference in low-density lipoprotein (LDL) levels was borderline significant (*p* = 0.055). No significant differences were observed in vitamin D, ferritin, iron, or total iron-binding capacity (all *p* > 0.05) (Table 3).

### 2.4. AGE-SAF Across Study Groups and Inflammatory Indices

**Figure 1:**
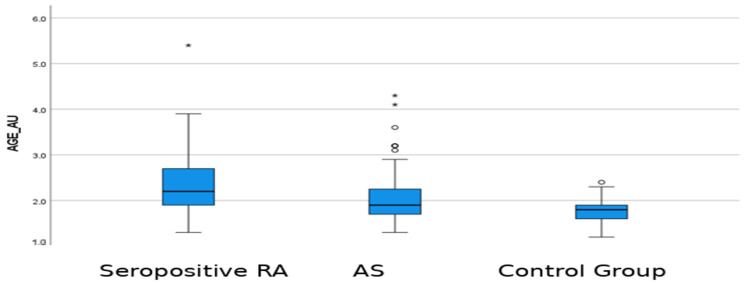
Distribution of AGE-SAF levels among seropositive RA patients, AS patients, and healthy controls. Box plots show medians, interquartile ranges, whiskers, and outlying values. The figure is intended as descriptive visualization; confounder-adjusted between-group estimates are reported in Section 2.8. Circles indicate outlying values. * *p* < 0.05; ** *p* < 0.01.

There was a significant difference in AGE-SAF levels among the groups (*p* < 0.001). In pairwise comparisons, the AGE-SAF value in the seropositive RA group was significantly higher than both the control group (adj. *p* < 0.001) and the AS group (adj. *p* = 0.001). Additionally, the AGE-SAF value in the AS group was significantly higher than that of the control group (adj. *p* < 0.001). Among the advanced glycation and inflammation-related indices, AGE-SAF values were higher in the seropositive RA group (*p* < 0.001). In contrast, the HALP score and CALLY index were higher in the AS group (both *p* < 0.001). No significant difference was observed between the groups in terms of SII values (*p* = 0.200) (Table 4, Figure 1). Figure 1 is presented for descriptive visualization, whereas interpretation of between-group differences relies primarily on the adjusted analyses reported below. In subgroup analyses according to medication class, AGE-SAF levels did not differ significantly within either the RA or AS group (RA Kruskal–Wallis *p* = 0.251; AS Kruskal–Wallis *p* = 0.663). Several subgroups contained fewer than five patients, and these results should be interpreted cautiously.

### 2.5. Correlation Analyses

In the seropositive RA group, a weak positive correlation was observed between AGE-SAF and steroid dose (r = 0.242, *p* = 0.030), a weak-to-moderate positive correlation with creatinine (r = 0.312, *p* = 0.005), and a moderate positive correlation with DAS28 (r = 0.499, *p* < 0.001). In the AS group, a moderate positive correlation was observed only between AGE-SAF and BASDAI (r = 0.360, *p* = 0.001). No significant correlations were found with disease duration, composite inflammatory indices, CRP, ESR, albumin, lipid variables, or HbA1c in either group (Table 5). After Benjamini–Hochberg false discovery rate (FDR) correction, only DAS28 in RA and BASDAI in AS remained significant. The full FDR-adjusted *q*-values are provided in Appendix A Table A1, Table A2, Table A3 and Table A4.

### 2.6. Clinical Subgroup Analyses

Higher AGE-SAF values were observed in patients with active disease in both groups (RA: *p* = 0.011; AS: *p* = 0.003). In the RA group, AGE-SAF levels were also associated with coronary artery disease (*p* = 0.004), hyperlipidemia (*p* = 0.003), atherosclerosis (*p* = 0.001), and cerebrovascular disease (*p* = 0.005). In the AS group, AGE-SAF levels were significantly associated with overall comorbidity burden (*p* = 0.016), coronary artery disease (*p* = 0.019), hypertension (*p* = 0.039), hyperlipidemia (*p* = 0.029), and atherosclerosis (*p* < 0.001). Steroid use comparison was not applicable in RA because all RA patients received corticosteroids, and no statistically significant association with steroid use was observed in AS. Major organ involvement was not significantly associated with AGE-SAF in RA and was not applicable in AS because no AS patients had major organ involvement. Osteoporosis was not significantly associated with AGE-SAF in RA; AS analyses for diabetes mellitus, osteoporosis, and cerebrovascular disease were treated as descriptive because of very small positive subgroups. After FDR correction, active disease, coronary artery disease, hyperlipidemia, atherosclerosis, and cerebrovascular disease remained significant in RA. In AS, active disease, overall comorbidity burden, coronary artery disease, hypertension, hyperlipidemia, and atherosclerosis met the FDR threshold; exploratory FDR calculations for diabetes mellitus and osteoporosis also met the threshold but were based on very small positive subgroups and should not be interpreted as robust inferential findings. The FDR-adjusted *q*-values for all comparisons are provided in Appendix A Table A1, Table A2, Table A3 and Table A4. Detailed subgroup comparisons are presented in Table 6.

### 2.7. Exploratory Disease-Specific Multivariable Models

As exploratory supportive analyses, disease-specific multivariable linear regression models were constructed for AGE-SAF. In the RA group, the model was overall statistically significant (*p* = 0.001) and explained 30.2% of the variance in AGE-SAF values (R^2^ = 0.302; adjusted R^2^ = 0.223). DAS28 (B = 0.115, β = 0.389, *p* = 0.012) and coronary artery disease (B = 0.421, β = 0.261, *p* = 0.033) were independently associated with AGE-SAF, whereas steroid dose, creatinine, disease activity, hyperlipidemia, atherosclerosis, and cerebrovascular disease were not (Table 7). Because these within-disease models were based on variable selection from univariate screening, they should be interpreted as exploratory and supportive rather than primary analyses.

In the AS group, the exploratory multivariable model was overall statistically significant (F = 5.197, *p* < 0.001) and explained 32.7% of the variance in AGE-SAF levels (R^2^ = 0.327; adjusted R^2^ = 0.264). Atherosclerosis showed a strong and independent positive association with AGE-SAF (B = 1.034, β = 0.499, *p* < 0.001), whereas steroid dose, BASDAI score, disease activity, hyperlipidemia, osteoporosis, and cerebrovascular disease were not independently associated (Table 8). As above, these disease-specific models are presented as exploratory and supportive analyses; the primary inference regarding between-group differences is based on the adjusted and sensitivity analyses reported below. To reduce the risk of overfitting, pre-specified parsimonious multivariable models were additionally fitted using only a small number of clinically relevant variables. In the reduced RA model (AGE-SAF ~ age + sex + BMI + DAS28 + coronary artery disease; n = 80, 5 covariates), DAS28 (β = 0.085, *p* = 0.005) and coronary artery disease (β = 0.495, *p* = 0.040) remained independently associated with AGE-SAF. In the reduced AS model (AGE-SAF ~ age + sex + BMI + BASDAI + atherosclerosis; n = 83, 5 covariates), BASDAI (β = 0.070, *p* = 0.001) and atherosclerosis (β = 0.797, *p* = 0.001) remained independently associated with AGE-SAF. These parsimonious models yielded conclusions consistent with the broader regressions while reducing the risk of model instability.

### 2.8. Confounder-Adjusted Between-Group Comparison and Sensitivity Analyses

Because the RA group was significantly older than the AS and control groups and had higher BMI and a different sex distribution, a multivariable linear regression analysis was performed with AGE-SAF as the dependent variable and study group, age, sex, and BMI as covariates, using robust (HC3) standard errors. After adjustment, AGE-SAF remained significantly higher in both RA (β = 0.440, 95% CI 0.298–0.583, *p* < 0.001) and AS (β = 0.304, 95% CI 0.183–0.425, *p* < 0.001) compared with controls. However, the adjusted difference between RA and AS was no longer statistically significant (β = 0.136, 95% CI −0.051 to 0.323, *p* = 0.154). When current glucocorticoid use was additionally included in the model, the RA-AS coefficient further attenuated (β = 0.114, *p* = 0.633); when diabetes mellitus, hypertension, and hyperlipidemia were further added, the RA-AS coefficient was essentially null (β = 0.060, *p* = 0.816) (Table 9).

To examine whether background hyperglycemia could explain the findings, two pre-specified sensitivity analyses were performed. Exclusion of patients with diabetes mellitus did not materially change the adjusted RA-AS difference (β = 0.152, 95% CI −0.033 to 0.337, *p* = 0.108), and exclusion of patients with HbA1c ≥ 6.5% yielded a similar result (β = 0.172, 95% CI −0.003 to 0.347, *p* = 0.054). These sensitivity analyses support the robustness of the confounder-adjusted findings.

### 2.9. Exploratory Discriminative Performance of AGE-SAF

**Figure 2:**
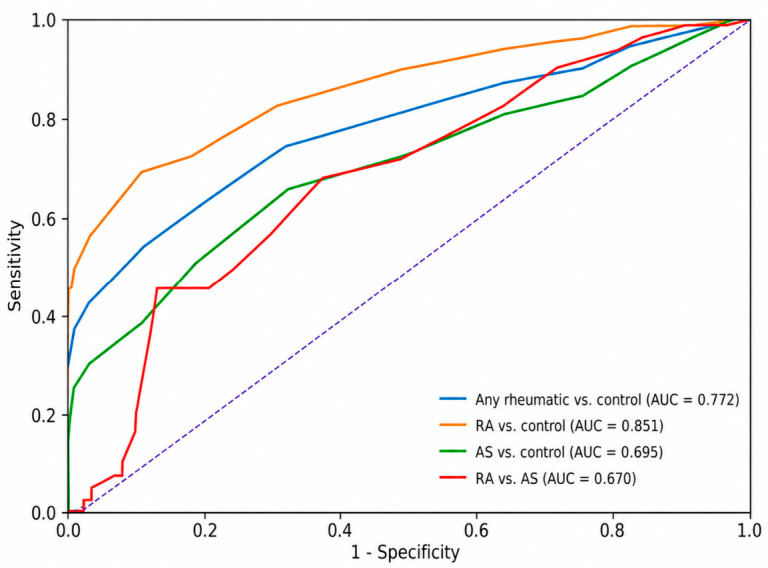
Receiver operating characteristic curves for AGE-SAF in the pairwise group comparisons. The dashed diagonal line represents the reference line for no discriminative ability.

In response to the reviewers’ request to explore the biomarker potential of AGE-SAF, receiver operating characteristic (ROC) curves and multivariable logistic regression models adjusted for age, sex, and BMI were constructed for pairwise outcomes. Because of the cross-sectional case–control design, AGE-SAF thresholds are described as sample-derived exploratory discriminative cut-offs rather than clinically validated diagnostic thresholds. For any rheumatic disease versus controls, the Youden-optimal AGE-SAF cut-off was 2.1 AU (AUC = 0.772, 95% CI 0.720–0.818; sensitivity 52.8%, specificity 88.0%; adjusted OR 16.51, 95% CI 6.99–38.99). For RA versus controls, the optimal cut-off was 2.1 AU (AUC = 0.851, 95% CI 0.794–0.900; sensitivity 67.5%, specificity 88.0%; adjusted OR 62.69, 95% CI 15.27–257.31). For AS versus controls, the optimal cut-off was 1.9 AU (AUC = 0.695, 95% CI 0.616–0.766; sensitivity 65.1%, specificity 66.5%; adjusted OR 16.70, 95% CI 5.74–48.61). For RA versus AS, AGE-SAF showed weak-to-moderate discriminative performance (AUC = 0.670, 95% CI 0.582–0.753); in the adjusted logistic model AGE-SAF was not an independent discriminator (OR 1.56, 95% CI 0.76–3.21, *p* = 0.225) (Table 10, Figure 2).

### 2.10. Propensity Score Sensitivity Analysis (RA Versus AS)

**Figure 3:**
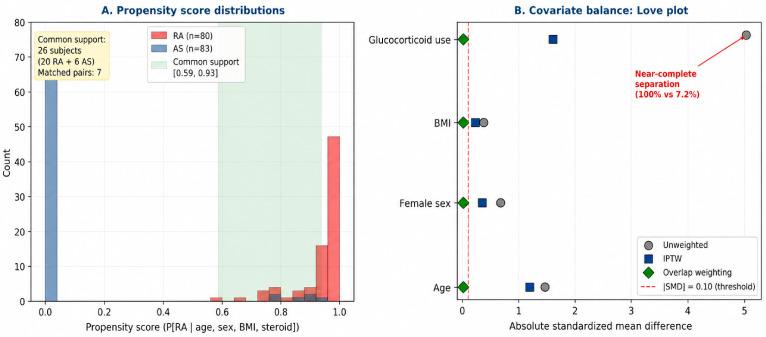
Propensity score distributions and covariate balance (Love plot) for the RA versus AS comparison, illustrating the positivity violation for glucocorticoid use and the region of common support used in the overlap-weighted sensitivity analysis.

To address reviewer concerns regarding baseline imbalance in age, sex, BMI, and glucocorticoid use between the RA and AS groups, propensity score analyses were performed. A propensity model based on age, sex, BMI, and current glucocorticoid use was fitted; however, because glucocorticoid use was nearly universal in RA (100%) and uncommon in AS (7.2%), marked propensity separation was observed. The region of common support contained only 26 patients (20 RA and 6 AS), and 1:1 caliper matching retained only 7 matched pairs. Stabilized inverse-probability-of-treatment weighting did not achieve acceptable balance for glucocorticoid use, whereas overlap weighting achieved excellent balance for age, sex, and BMI but, as expected, could not fully resolve the positivity problem created by near-complete separation in glucocorticoid exposure. In the overlap-weighted analysis—interpreted as restricted to the subpopulation with genuine covariate overlap—the RA-AS difference in AGE-SAF was not statistically significant (β = 0.161, *p* = 0.293), consistent with the primary confounder-adjusted regression. Accordingly, propensity score analyses are reported as sensitivity analyses only and were not used for primary inference (Figure 3).

### 2.11. Multiple-Testing Correction for Exploratory Analyses

To reduce the false-positive risk inherent to multiple exploratory comparisons, Benjamini–Hochberg false discovery rate correction was applied to the correlation and subgroup analyses. In the RA group, only the correlation between AGE-SAF and DAS28 remained significant after correction. In the AS group, only the correlation with BASDAI remained significant. Among the subgroup comparisons, the associations that remained significant after correction were active disease, coronary artery disease, hyperlipidemia, atherosclerosis, and cerebrovascular disease in RA, and active disease, overall comorbidity burden, coronary artery disease, hypertension, hyperlipidemia, and atherosclerosis in AS. Exploratory FDR calculations for diabetes mellitus and osteoporosis in AS also met the threshold but were based on very small positive subgroups and should be interpreted with extreme caution. The full FDR-adjusted *q*-values are provided in Appendix A Table A1, Table A2, Table A3 and Table A4 (four sheets: Table A1 RA correlations, Table A2 AS correlations, Table A3 RA subgroups, Table A4 AS subgroups).

## 3. Discussion

In the present study, AGE-SAF levels were significantly higher in both RA and AS compared with healthy controls. After adjustment for age, sex, and BMI, both patient groups remained significantly higher than controls; however, the adjusted difference between RA and AS was no longer statistically significant. This indicates that a substantial part of the crude between-disease difference is attributable to baseline imbalance rather than a robust disease-specific effect.

This interpretation was supported by several additional analyses. Excluding patients with diabetes mellitus or HbA1c ≥ 6.5% did not materially change the adjusted RA-AS estimate. Propensity score approaches were also explored, but the near-universal glucocorticoid exposure in RA and the very low exposure in AS created marked propensity separation and poor common support. In the overlap-weighted sensitivity analysis, the RA-AS difference remained non-significant. Together, these results suggest that the crude RA > AS contrast in AGE-SAF is largely driven by confounding from age, sex, BMI, and treatment exposure.

The finding that both RA and AS exhibited higher AGE-SAF than controls remain biologically plausible. Chronic inflammation, oxidative stress, vascular injury, and cardiometabolic comorbidity can all promote AGE formation and AGE-RAGE signaling [3,4,6,8,9,10,19]. In our cohort, RA patients also had higher CRP and ESR, lower albumin, more comorbidity, and substantially greater glucocorticoid exposure, each of which may contribute to cumulative tissue AGE burden.

From a mechanistic standpoint, AGEs may provide a critical link between chronic inflammation, metabolic dysregulation, and tissue damage. AGEs are formed through non-enzymatic glycation and oxidation of proteins and lipids under conditions of oxidative stress and hyperglycemia [3,8]. Chronic inflammation may further drive AGE formation through increased reactive oxygen species generation and enhanced glycoxidation pathways [8,19]. Once formed, AGEs interact with the RAGE, leading to activation of intracellular signaling pathways—particularly NF-κB—which may amplify the production of pro-inflammatory cytokines such as IL-1, IL-6, and TNF-α [4,6,8]. This AGE–RAGE–NF-κB axis establishes a self-perpetuating cycle of inflammation and oxidative stress which may contribute to endothelial dysfunction, vascular injury, and progressive tissue damage [4,6,8,9].

Treatment-related factors also appear to play a significant role. Glucocorticoid exposure deserves particular emphasis. In routine practice, systemic glucocorticoids are used more frequently in RA than in axial spondyloarthritis, and the present dataset reflected this pattern very strongly [20,21,22]. Because glucocorticoid exposure was nearly universal in RA but uncommon in AS, its independent contribution could not be disentangled from disease-group effects. This is a major limitation of the between-disease comparison and likely explains part of the crude difference in AGE-SAF.

AGE-SAF showed exploratory discriminative ability when patient groups were compared with controls, with the strongest performance observed for RA versus control. However, discrimination between RA and AS was only weak to moderate and disappeared after covariate adjustment. Because the study used a cross-sectional case–control design, the reported cut-offs should be interpreted as sample-derived exploratory thresholds rather than clinically validated diagnostic values.

The correlation and subgroup analyses also support a cautious interpretation. AGE-SAF was not significantly correlated with CRP or ESR, and after FDR correction only DAS28 in RA and BASDAI in AS remained significant among the correlation analyses. Similarly, only a subset of the nominal comorbidity associations remained after FDR correction. Taken together, these findings suggest that AGE-SAF is better viewed as a marker of cumulative disease-related and vascular–metabolic burden than as a surrogate of short-term inflammatory activity.

The exploratory disease-specific multivariable models were consistent with this interpretation: disease activity and coronary artery disease were independently associated with AGE-SAF in RA, whereas atherosclerosis dominated in AS. These within-disease models may be useful for hypothesis generation, but they should not be weighted more heavily than the primary between-group adjusted analyses because their variable selection strategy carries an overfitting risk [6,8,12,19,23,24].

However, these findings should be interpreted with caution. The cross-sectional design precludes causal inference, and differences in age, sex distribution, treatment exposure, and comorbidity burden between groups may have contributed to residual confounding.

This study has several limitations. Its single-center cross-sectional design limits causal inference and generalizability. Important baseline imbalances existed between groups, especially for age, sex, BMI, and glucocorticoid exposure. Although these imbalances were addressed through multivariable adjustment, diabetes/HbA1c sensitivity analyses, and propensity score approaches, the near-complete separation in glucocorticoid use created a positivity violation that prevented stable matched estimates. In addition, common cardiometabolic comorbidities were retained in the final analytical dataset to reflect real-world clinical burden, which should be considered when interpreting protocol-to-dataset consistency. SAF provides a non-invasive measure of overall tissue AGE accumulation but does not distinguish specific AGE subtypes. Key metabolic indicators such as fasting insulin, HOMA-IR, and diabetes duration were not available from the routine clinical workflow from which the dataset was drawn; fasting glucose and HbA1c provided only partial glycemic characterization. These additional metabolic measures represent a priority for future prospective studies. The cross-sectional case–control design also does not support conclusions about drug class efficacy or drug combination effects on symptom improvement, and these questions should be addressed in longitudinal studies. Finally, the ROC-derived cut-offs are exploratory and require external prospective validation.

Despite these limitations, the study provides a comprehensive evaluation of tissue AGE accumulation across two major inflammatory rheumatic diseases using a non-invasive and reproducible method and integrates clinical, inflammatory, metabolic, and comorbidity-related parameters within a single analytical framework. The consistent elevation of AGE-SAF versus controls supports its potential relevance as a non-invasive marker of cumulative disease-related burden.

## 4. Materials and Methods

### 4.1. Study Design and Ethics

This observational cross-sectional case–control study was conducted at the rheumatology outpatient clinic of Bursa City Hospital (Bursa, Türkiye) to evaluate tissue AGE accumulation and its associations with clinical characteristics, laboratory parameters, inflammatory indices, and comorbidities in patients with inflammatory rheumatic diseases.

The study was conducted in accordance with the Declaration of Helsinki and approved by the Bursa City Hospital Clinical Research Ethics Committee (Approval No: 2026-2-10; Date: 21 January 2026). Written informed consent was obtained from all participants prior to enrollment. The study was registered at ClinicalTrials.gov (Identifier: NCT07329556).

### 4.2. Participants

The study population comprised 80 patients with seropositive RA, 83 patients with AS, and 200 healthy controls. Recruitment continued throughout the predefined screening window, yielding the final analytical sample used in the present report. RA was classified according to the 2010 American College of Rheumatology/European League Against Rheumatism criteria [1], and AS according to the Assessment of SpondyloArthritis International Society classification criteria [25].

Participants aged 18 years or older with a confirmed diagnosis of RA or AS were eligible. Healthy controls had no history of inflammatory, autoimmune, or systemic chronic rheumatic disease. The final analytical dataset reflected routine outpatient clinical sampling; therefore, common cardiometabolic comorbidities such as diabetes mellitus, hypertension, and hyperlipidemia were retained rather than excluded, because they were part of the cumulative clinical burden under study. Exclusion criteria were active infection within the previous two weeks, active malignancy, major surgery within the previous three months, chronic inflammatory diseases other than RA or AS, and inability to undergo AGE-SAF measurement. Current smokers were excluded. Only participants who had never smoked or had quit smoking at least 6 months before enrollment were included.

### 4.3. Clinical and Laboratory Variables

Demographic and clinical data were recorded for all participants, including age, sex, body mass index (BMI), disease duration, medication use, and comorbidities. Comorbidities included hypertension, hyperlipidemia, coronary artery disease, cerebrovascular disease, osteoporosis and other chronic systemic diseases. Clinical and laboratory data were obtained from routine outpatient evaluations. The treatments used in both patient groups reflected standard-of-care regimens administered at internationally recommended clinical doses. With respect to pharmaceutical form and route of administration, NSAIDs and conventional synthetic DMARDs (methotrexate, sulfasalazine, leflunomide, hydroxychloroquine) were administered orally; glucocorticoids (prednisolone) were administered orally; anti-TNF agents were given subcutaneously (adalimumab, etanercept, certolizumab, golimumab) or intravenously (infliximab) according to their approved routes; the IL-17 inhibitor was administered subcutaneously; the JAK inhibitor was administered orally; tocilizumab was administered intravenously or subcutaneously; and rituximab was administered intravenously. Regarding daily administration, prednisolone was given once daily; in the RA group, the steroid-dose distribution was 2.5 mg in 2 patients (2.5%), 5 mg in 77 patients (96.3%), and 10 mg in 1 patient (1.3%), whereas in the AS group 6 of 83 patients (7.2%) received 5 mg daily prednisolone. Conventional synthetic DMARDs were administered at conventional recommended doses (methotrexate 10–25 mg weekly; sulfasalazine 2–3 g/day; hydroxychloroquine 200–400 mg/day; leflunomide 10–20 mg/day). Biologic and targeted synthetic agents were given at manufacturer-approved doses and intervals, and NSAIDs at standard recommended doses. Because treatment duration and exact regimen varied across patients, drug dose was not analyzed as a continuous variable except for the steroid–dose correlation reported in Table 5.

Disease activity in RA was assessed using the DAS28, which incorporates tender and swollen joint counts (TJC28, SJC28), an acute-phase reactant (ESR or CRP), and the patient’s global assessment. DAS28 scores range from 0 to 10, with higher values indicating greater disease activity. Disease activity categories were defined as remission (<2.6), low (2.6–3.2), moderate (3.2–5.1), and high (>5.1) [26,27].

Disease activity in AS was evaluated using the BASDAI, a patient-reported index consisting of six items assessing fatigue, spinal pain, peripheral joint symptoms, enthesitis, and morning stiffness. Each item is scored on a 10-point visual analog scale, and the final score ranges from 0 to 10, with higher scores indicating greater disease activity. A BASDAI score ≥ 4 was considered indicative of active disease [28]. The Turkish version of BASDAI has been validated and shown to be reliable [29].

Laboratory parameters obtained from routine clinical evaluations included CRP, ESR, albumin, hemoglobin, lymphocyte count, neutrophil count, platelet count, glucose, lipid profile (LDL, HDL, triglycerides, total cholesterol), creatinine, ALT, AST, vitamin B12, vitamin D, ferritin, iron, and total iron-binding capacity.

### 4.4. AGE-SAF Measurement

Tissue AGE accumulation was assessed non-invasively using SAF with the AGE Reader™ device (DiagnOptics Technologies B.V., Groningen, The Netherlands). Measurements were performed on the volar side of the non-dominant forearm under standardized conditions by trained personnel. Areas with visible skin lesions, scars, or pigmentation abnormalities were avoided. Participants were instructed not to apply creams or lotions to the measurement site before assessment. Three consecutive measurements were obtained, and the mean value was recorded as AGE-SAF.

Composite inflammatory indices were calculated using standard formulas. The SII was calculated as platelet count × neutrophil count/lymphocyte count. The HALP score was calculated as hemoglobin × albumin × lymphocyte count/platelet count. The CALLY index was calculated as (albumin × lymphocyte count)/CRP [16,17,18].

### 4.5. Statistical Analysis

Statistical analysis was performed using the SPSS v31 software (IBM Corp., Armonk, NY, USA) and R version 4.3.0 (R Core Team, Vienna, Austria) for the propensity score, ROC, and false discovery rate analyses. Descriptive data are presented as number and percentage for categorical variables and as mean ± standard deviation or median (minimum–maximum) for numerical variables. Normality was assessed using the Kolmogorov–Smirnov test and histogram evaluation.

Comparisons between groups were performed using the chi-square test or Fisher’s exact test for categorical variables, and Student’s *t*-test or Mann–Whitney U test for continuous variables, as appropriate. For comparisons involving more than two groups, the Kruskal–Wallis test was used, followed by Bonferroni-corrected post hoc analysis when necessary. Correlations between numerical variables were evaluated using Spearman’s correlation analysis.

To address confounding in the between-group comparison of AGE-SAF, a multivariable linear regression model was fitted with AGE-SAF as the dependent variable and study group, age, sex, and BMI as covariates, using robust (HC3) standard errors. Pre-specified sensitivity analyses additionally adjusted for current glucocorticoid use and selected metabolic comorbidities (diabetes mellitus, hypertension, hyperlipidemia) and repeated the analysis after excluding patients with diabetes mellitus and those with HbA1c ≥ 6.5%. The exploratory discriminative performance of AGE-SAF was described using ROC analysis with Youden-optimal cut-offs, and logistic regression models adjusted for age, sex, and BMI were used to estimate covariate-adjusted odds ratios. Because of the cross-sectional case–control design, these thresholds were treated as sample-derived and exploratory.

To further address baseline imbalance between the RA and AS groups, propensity score analyses (1:1 caliper matching, stabilized inverse-probability-of-treatment weighting, and overlap weighting) were performed as sensitivity analyses using age, sex, BMI, and current glucocorticoid use. Covariate balance was assessed using standardized mean differences, with values < 0.10 considered acceptable. Disease-specific multivariable linear regression models were additionally fitted as exploratory supportive analyses. Multiple-testing adjustment for the exploratory correlation and subgroup analyses was performed using the Benjamini–Hochberg false discovery rate procedure. A two-sided *p* value < 0.05 was considered statistically significant.

## 5. Conclusions

In conclusion, AGE-SAF levels are significantly elevated in patients with inflammatory rheumatic diseases compared with healthy controls, and this elevation persists after adjustment for age, sex, and BMI. Although crude AGE-SAF values were higher in RA than in AS, the between-disease difference attenuated and was no longer statistically significant after adjustment for demographic and treatment-related factors. AGE-SAF therefore appears to reflect cumulative disease-related and vascular–metabolic burden rather than a disease-specific mechanism or a direct marker of short-term inflammatory activity.

These findings support the potential clinical relevance of AGE-SAF as a non-invasive indicator of cumulative disease burden. However, the ROC-derived thresholds reported here are exploratory and sample-derived, and prospective studies in independent cohorts are required before clinical application.

## Figures and Tables

**Table 1:** Demographic Characteristics of the Study Groups.

		Seropositive RA	AS	Control Group	*p*
Age		58.35 ± 12.49; 59 (25–80)	41.92 ± 10.78; 42 (21–75)	40.51 ± 11.13; 41 (18–74)	<0.001 ^#^
Sex	Male	23 (28.7%)	48 (57.8%)	98 (49.0%)	0.001 ^¥^
	Female	57 (71.3%)	35 (42.2%)	102 (51.0%)	
BMI		29.26 ± 4.94; 28.96 (21.1–44.8)	27.68 ± 4.99; 27.34 (17.6–41.3)	26.88 ± 4.24; 26.12 (18.7–41.9)	0.001 ^#^

^¥^ Chi-square test; ^#^ Kruskal–Wallis test.

**Table 2:** Clinical Parameters.

Parameter	Seropositive RA (n = 80)	AS (n = 83)		*p*
Disease duration (years) Mean ± SD; Median (Min–Max)	6.96 ± 5.51; 5 (0.1–17)	5.80 ± 5.21; 3 (1–18)		0.147 *
BASDAI Mean ± SD; Median (Min–Max)	—	3.42 ± 2.25; 2.30 (1.0–8.9)		—
DAS28 Mean ± SD; Median (Min–Max)	3.07 ± 2.13; 2.10 (1.0–8.3)	—		
RF (IU/mL) Mean ± SD; Median (Min–Max)	84.46 ± 65.82; 67 (0–305)	—		
Anti-CCP (U/mL) Mean ± SD; Median (Min–Max)	364.57 ± 170.97; 500 (0–500)	—		
Disease activity n (%)	Inactive	45 (56.3%)	46 (55.4%)	0.915 ^¥^
	Active	35 (43.8%)	37 (44.6%)	
Medications used n (%)	NSAIDs	0 (0.0%)	22 (26.5%)	<0.001 ^¥^
	Conventional therapy	62 (77.5%)	9 (10.8%)	
	Anti-TNF	7 (8.8%)	47 (56.6%)	
	JAK inhibitor therapy	4 (5.0%)	1 (1.2%)	
	IL-17 inhibitor	0 (0.0%)	3 (3.6%)	
	Rituximab	1 (1.3%)	0 (0.0%)	
	Initial therapy	0 (0.0%)	1 (1.2%)	
	Tocilizumab	6 (7.5%)	0 (0.0%)	
Steroid use n (%)	No	0 (0.0%)	77 (92.8%)	<0.001 ^¥^
	Yes	80 (100.0%)	6 (7.2%)	
Steroid dose (mg/day) n (%)	0	0 (0.0%)	77 (92.8%)	<0.001 ^¥^
	2.5 mg	2 (2.5%)	0 (0.0%)	
	5 mg	77 (96.3%)	6 (7.2%)	
	10 mg	1 (1.3%)	0 (0.0%)	
Major organ involvement n (%)	No	74 (92.5%)	83 (100.0%)	0.013 ^¥^
	Yes	6 (7.5%)	0 (0.0%)	
HLA-B27	Positivity	—	59 (72.0%)	-
	Negativity	—	23 (28.0%)	
Type of involvement	Axial	—	77 (93.9%)	-
	Peripheral	—	5 (6.1%)	
History of tuberculosis	Yes	—	1 (16.7%)	-
	No	—	5 (83.3%)	
Comorbidity n (%)	Yes	61 (76.3%)	40 (48.2%)	<0.001 ^¥^
	No	19 (23.8%)	43 (51.8%)	
	Diabetes Mellitus	12 (15.0%)	4 (4.8%)	0.029 ^¥^
	Coronary artery disease	15 (18.8%)	9 (10.8%)	0.154 ^¥^
	Hypertension	45 (56.3%)	19 (22.9%)	<0.001 ^¥^
	Hyperlipidemia	18 (22.5%)	7 (8.4%)	0.013 ^¥^
	Thyroid disease	0 (0.0%)	1 (1.2%)	1.000 ^¥^
	Chronic kidney disease	1 (1.3%)	0 (0.0%)	0.491 ^¥^
	Other comorbidities	24 (30.0%)	26 (31.3%)	0.854 ^¥^
Osteoporosis n (%)	No	61 (76.3%)	80 (96.4%)	<0.001 ^¥^
	Yes	19 (23.8%)	3 (3.6%)	
Atherosclerosis n (%)	No	66 (82.5%)	76 (91.6%)	0.084 ^¥^
	Yes	14 (17.5%)	7 (8.4%)	
Cerebrovascular disease n (%)	No	74 (92.5%)	81 (97.6%)	0.163 ^¥^
	Yes	6 (7.5%)	2 (2.4%)	

^¥^ Chi-square test; * Mann–Whitney U Test.

**Table 3:** Laboratory Parameters in Seropositive RA and AS Patients.

Parameter	Seropositive RA Mean ± SD; Median (Min–Max)	AS Mean ± SD; Median (Min–Max)	*p* *
CRP (mg/L)	7.70 ± 9.47; 4.10 (0.3–49.93)	5.22 ± 8.14; 2.23 (0.5–49)	0.032
ESR (mm/h)	18.22 ± 16.31; 12.50 (2–75)	10.58 ± 8.74; 8 (2–37)	0.002
Albumin (g/L)	43.17 ± 2.98; 43.3 (34.5–49.6)	44.57 ± 3.06; 44.8 (31–51.3)	0.006
Lymphocyte (10^9^/L)	2.21 ± 0.89; 1.95 (0.61–6.11)	2.66 ± 0.84; 2.46 (1.16–5.21)	<0.001
Neutrophil (10^9^/L)	4.35 ± 1.89; 4.09 (1.38–9.76)	4.70 ± 1.90; 4.64 (1.73–14.33)	0.200
Hemoglobin (g/dL)	13.10 ± 1.50; 12.0 (10–16.7)	14.8 ± 1.6; 14.7 (10.9–17.3)	<0.001
Platelet (10^9^/L)	280.35 ± 75.99; 259.5 (126–499)	285 ± 74.82; 276 (169–587)	0.693
Glucose (mg/dL)	100.49 ± 39.48; 91 (64.4–337)	96.47 ± 26.79; 89 (74–200)	0.726
ALT (U/L)	27.76 ± 36.45; 19 (5–301)	24.70 ± 15.39; 20 (8–109)	0.759
AST (U/L)	24.19 ± 18.10; 19.5 (8–130)	19.98 ± 6.56; 19 (8–50.0)	0.495
BUN (mg/dL)	15.63 ± 7.80; 14.2 (7.0–66)	14.09 ± 3.99; 14 (6.3–26)	0.293
Creatinine (mg/dL)	0.77 ± 0.21; 0.73 (0.52–1.57)	0.79 ± 0.15; 0.77 (0.39–1.17)	0.041 *
LDL (mg/dL)	119.46 ± 36.28; 113 (50–266)	107.78 ± 31.73; 104 (47–210)	0.055
HDL (mg/dL)	55.57 ± 16.71; 54 (20–113)	46.95 ± 14.81; 43.5 (23–99)	<0.001
Triglycerides (mg/dL)	161.28 ± 151.14; 128 (39–1304)	146.66 ± 99.18; 112 (30–606)	0.366
Total cholesterol (mg/dL)	207.58 ± 54.62; 197 (117–486)	182.64 ± 42.30; 187 (95–323)	0.001 **
HbA1c (%)	5.83 ± 1.11; 5.68 (4.63–14)	5.66 ± 0.95; 5.46 (4.47–9.7)	0.024
B12 (pg/mL)	463.88 ± 381.20; 382 (95–3407)	373.45 ± 161.20; 354 (143–1183)	0.039
Vitamin D (ng/mL)	23.93 ± 12.03; 23.75 (1–58.60)	20.69 ± 10.59; 22.30 (3–63.7)	0.067
Ferritin (ng/mL)	90.89 ± 105.68; 64.95 (6.73–688)	84.66 ± 77.43; 61 (0.80–455)	0.827
Iron (µg/dL)	73.06 ± 37.51; 64.5 (17–225)	78.19 ± 39.50; 70 (11–237)	0.313
Total iron-binding capacity (µg/dL)	277.77 ± 75.53; 271 (107.2–480)	264.56 ± 71.41; 251 (103.3–442)	0.153

* Mann–Whitney U test; ** Student’s *t*-test.

**Table 4:** AGE-SAF levels across study groups and inflammation-related indices in patient groups.

	Seropositive RA	AS	Control Group	*p*
	Mean ± SD; Median (Min–Max)	Mean ± SD; Median (Min–Max)	Mean ± SD; Median (Min–Max)	
AGE-SAF	2.37 ± 0.63; 2.20 (1.3–5.4)	2.07 ± 0.58; 1.90 (1.3–4.3)	1.73 ± 0.26; 1.80 (1.2–2.4)	<0.001 ^#^
SII	647.90 ± 495.47; 546.41 (79.8–2856.6)	566.09 ± 359.28; 446.1 (106.3–1758.8)		0.200 *
HALP score	4.73 ± 2.14; 4.09 (1.24–9.22)	6.64 ± 4.28; 5.96 (1.82–35.85)		<0.001 *
CALLY index	0.89 ± 1.13; 0.40 (0.02–5.19)	2.21 ± 2.59; 1.28 (0.04–13.14)		<0.001 *

^#^ Kruskal–Wallis test; * Mann–Whitney U test.

**Table 5:** Relationship Between AGE-SAF and Clinical and Laboratory Parameters.

Parameter	Seropositive RA		AS	
	AGE-SAF		AGE-SAF	
	r	*p*	r	*p*
Disease duration (years)	−0.147	0.192	−0.070	0.532
Steroid dose (mg/day)	0.242	0.030	−0.023	0.834
BASDAI			0.360	0.001
DAS28	0.499	<0.001		
RF (IU/mL)	0.062	0.583		
Anti-CCP (U/mL)	−0.019	0.865		
SII	0.126	0.266	0.079	0.480
HALP score	−0.001	0.995	−0.043	0.702
CALLY index	−0.122	0.280	−0.084	0.451
CRP (mg/L)	0.117	0.300	0.000	0.997
ESR (mm/h)	0.182	0.106	0.095	0.392
Albumin (g/L)	0.042	0.714	−0.124	0.262
Lymphocyte (10^9^/L)	−0.061	0.594	−0.003	0.975
Neutrophil (10^9^/L)	0.205	0.068	0.153	0.167
Hemoglobin (g/dL)	0.131	0.246	0.001	0.990
Platelet (10^9^/L)	0.042	0.713	−0.020	0.857
Glucose (mg/dL)	0.146	0.196	0.094	0.397
ALT (U/L)	0.132	0.244	−0.073	0.511
AST (U/L)	0.098	0.388	−0.032	0.775
BUN (mg/dL)	0.170	0.131	−0.038	0.736
Creatinine (mg/dL)	0.312	0.005	−0.066	0.551
LDL (mg/dL)	0.150	0.185	−0.111	0.317
HDL (mg/dL)	−0.169	0.133	−0.034	0.757
Triglycerides (mg/dL)	0.199	0.077	0.186	0.092
Total cholesterol (mg/dL)	0.127	0.263	0.034	0.762
HbA1c (%)	0.206	0.067	0.198	0.072

r: Spearman correlation coefficient; *p*: corresponding *p* value.

**Table 6:** Comparison of AGE-SAF levels according to clinical characteristics in seropositive RA and AS patients.

		Seropositive RA		AS	
		AGE-SAF Mean ± SD; Median (Min–Max)	*p*	AGE-SAF Mean ± SD; Median (Min–Max)	*p*
Disease activity	Inactive	2.28 ± 0.71; 2.10 (1.3–5.4)	0.011	1.94 ± 0.58; 1.80 (1.3–4.3)	0.003
	Active	2.49 ± 0.50; 2.50 (1.5–3.9)		2.23 ± 0.55; 2.00 (1.7–4.1)	
Steroid use	No	—	—	2.07 ± 0.58; 1.90 (1.3–4.3)	0.832
	Yes	2.37 ± 0.63; 2.20 (1.3–5.4)		2.05 ± 0.60; 1.95 (1.5–3.2)	
Major organ involvement	No	2.38 ± 0.65; 2.20 (1.3–5.4)	0.978	2.07 ± 0.58; 1.90 (1.3–4.3)	—
	Yes	2.30 ± 0.45; 2.30 (1.6–2.8)		—	
Comorbidity	No	2.16 ± 0.45; 2.10 (1.3–3.0)	0.136	1.92 ± 0.46; 1.90 (1.3–3.2)	0.016
	Yes	2.44 ± 0.67; 2.30 (1.5–5.4)		2.23 ± 0.65; 2.10 (1.5–4.3)	
Diabetes Mellitus	No	2.32 ± 0.63; 2.20 (1.3–5.4)	0.056	2.02 ± 0.52; 1.90 (1.3–4.3)	—
	Yes	2.66 ± 0.60; 2.55 (1.9–3.9)		2.95 ± 1.06; 2.85 (2.0–4.1)	
Coronary artery disease	No	2.27 ± 0.53; 2.10 (1.3–3.9)	0.004	2.02 ± 0.56; 1.90 (1.3–4.3)	0.019
	Yes	2.81 ± 0.84; 2.70 (2.1–5.4)		2.49 ± 0.60; 2.50 (1.7–3.6)	
Hypertension	No	2.20 ± 0.44; 2.10 (1.3–3.0)	0.067	1.96 ± 0.43; 1.90 (1.3–3.2)	0.039
	Yes	2.51 ± 0.72; 2.50 (1.6–5.4)		2.43 ± 0.83; 2.30 (1.5–4.3)	
Hyperlipidemia	No	2.26 ± 0.54; 2.10 (1.3–3.9)	0.003	2.01 ± 0.51; 1.90 (1.3–4.3)	0.029
	Yes	2.77 ± 0.77; 2.65 (1.8–5.4)		2.70 ± 0.89; 2.40 (1.7–4.1)	
Other comorbidities	No	2.37 ± 0.68; 2.20 (1.3–5.4)	0.524	1.98 ± 0.53; 1.90 (1.3–4.3)	0.065
	Yes	2.39 ± 0.50; 2.50 (1.5–3.3)		2.25 ± 0.65; 2.10 (1.5–4.1)	
Osteoporosis	No	2.42 ± 0.67; 2.30 (1.3–5.4)	0.227	2.05 ± 0.58; 1.90 (1.3–4.3)	—
	Yes	2.23 ± 0.47; 2.10 (1.5–3.3)		2.57 ± 0.35; 2.60 (2.2–2.9)	
Atherosclerosis	No	2.29 ± 0.64; 2.10 (1.3–5.4)	0.001	1.99 ± 0.50; 1.90 (1.3–4.3)	˂0.001
	Yes	2.76 ± 0.43; 2.75 (1.8–3.5)		2.94 ± 0.69; 2.90 (2.2–4.1)	
Cerebrovascular disease	No	2.32 ± 0.62; 2.20 (1.3–5.4)	0.005	2.06 ± 0.58; 1.90 (1.3–4.3)	—
	Yes	3.00 ± 0.49; 2.80 (2.6–3.9)		2.55 ± 0.50; 2.55 (2.2–2.9)	

Mann–Whitney U test.—Statistical comparison was not performed because of insufficient sample size in one or both subgroups.

**Table 7:** Multivariate Linear Regression Analysis for AGE-SAF in the Seropositive RA Group.

Variable	B	Beta	*p*
Constant	1.501		
Steroid dose (mg/day)	0.066	0.072	0.487
DAS28	0.115	0.389	0.012
Creatinine (mg/dL)	0.153	0.050	0.655
Disease activity	−0.173	−0.137	0.355
Coronary artery disease	0.421	0.261	0.033
Hyperlipidemia	0.238	0.158	0.208
Atherosclerosis	−0.095	−0.058	0.681
Cerebrovascular disease	0.436	0.183	0.149

B: unstandardized regression coefficient; Beta: standardized regression coefficient.

**Table 8:** Multivariate Linear Regression Analysis for AGE-SAF in the AS Group.

Variable	B	Beta	*p*
Constant	1.815		
Steroid dose (mg/day)	0.002	0.004	0.971
BASDAI	0.007	0.026	0.916
Disease activity	0.290	0.250	0.314
Hyperlipidemia	0.174	0.084	0.472
Osteoporosis	0.316	0.102	0.344
Atherosclerosis	1.034	0.499	<0.001
Cerebrovascular disease	−0.555	−0.148	0.226

B: unstandardized regression coefficient; Beta: standardized regression coefficient.

**Table 9:** Confounder-adjusted between-group comparison of AGE-SAF.

Comparison	β (Adjusted Difference)	95% CI Lower	95% CI Upper	*p*
RA vs. control (age + sex + BMI adj.)	0.440	0.298	0.583	<0.001
AS vs. control (age + sex + BMI adj.)	0.304	0.183	0.425	<0.001
RA vs. AS (age + sex + BMI adj.)	0.136	−0.051	0.323	0.154
RA vs. AS (+glucocorticoid use)	0.114	−0.355	0.584	0.633
RA vs. AS (+glucocorticoid + DM + HT + HLP)	0.060	−0.447	0.568	0.816

Values are regression coefficients (β) with robust (HC3) 95% confidence intervals.

**Table 10:** Exploratory discriminative performance of AGE-SAF (ROC and covariate-adjusted logistic regression).

Outcome	AUC (95% CI)	Cut-off (AU)	Sens.	Spec.	Adjusted OR (95% CI); *p*
Any rheumatic vs. control	0.772 (0.720–0.818)	2.1	52.8%	88.0%	16.51 (6.99–38.99) *p* < 0.001
RA vs. control	0.851 (0.794–0.900)	2.1	67.5%	88.0%	62.69 (15.27–257.31) *p* < 0.001
AS vs. control	0.695 (0.616–0.766)	1.9	65.1%	66.5%	16.70 (5.74–48.61) *p* < 0.001
RA vs. AS	0.670 (0.582–0.753)	2.5	45.0%	85.5%	1.56 (0.76–3.21) *p* = 0.225

AUC, area under the ROC curve; Sens., sensitivity at the Youden-optimal cut-off; Spec., specificity; adjusted OR, odds ratio from a logistic regression model including age, sex, and BMI. Thresholds are exploratory and sample-derived.

## Data Availability

The data presented in this study are available on reasonable request from the corresponding author. The data are not publicly available due to privacy and ethical restrictions.

## Abbreviations

The following abbreviations are used in this manuscript:

AGEs	Advanced glycation end products
AGE-SAF	Advanced glycation end products–skin autofluorescence
SAF	Skin autofluorescence
RAGE	Receptor for advanced glycation end products
NF-κB	Nuclear factor kappa B
RA	Rheumatoid arthritis
AS	Ankylosing spondylitis
DAS28	Disease Activity Score in 28 joints
BASDAI	Bath Ankylosing Spondylitis Disease Activity Index
CRP	C-reactive protein
ESR	Erythrocyte sedimentation rate
SII	Systemic immune-inflammation index
HALP	Hemoglobin–albumin–lymphocyte–platelet score
CALLY	C-reactive protein–albumin–lymphocyte index
BMI	Body mass index
LDL	Low-density lipoprotein
HDL	High-density lipoprotein
ALT	Alanine aminotransferase
AST	Aspartate aminotransferase
BUN	Blood urea nitrogen
RF	Rheumatoid factor
Anti-CCP	Anti-cyclic citrullinated peptide
TNF-α	Tumor necrosis factor alpha
IL-1	Interleukin-1
IL-6	Interleukin-6
NSAIDs	Nonsteroidal Anti-Inflammatory Drugs
Hemoglobin A1c	HbA1c

## Appendix A. Multiple-Testing-Corrected (FDR) Results

*p*-values are from Spearman’s rank correlation (for continuous associations) or from the Mann–Whitney U test (for between-subgroup comparisons of AGE-SAF). False discovery rate (FDR) was controlled at 5% using the Benjamini–Hochberg procedure, applied separately within each disease group and within each family of tests (correlations or subgroups). Rows shaded in green indicate *q* < 0.05 after FDR correction.

**Table A1:** RA—Spearman correlations of AGE-SAF (FDR-adjusted).

Parameter	n	Spearman r	*p* (Unadjusted)	*q* (BH-FDR)
Disease duration	80	−0.147	0.1922	0.4075
Steroid dose	80	0.242	0.0305	0.2538
DAS28	80	0.499	<0.001	<0.001
RF	80	0.062	0.5834	0.7070
Anti-CCP	80	−0.019	0.8652	0.9013
SII	80	0.126	0.2661	0.4111
HALP	80	−0.001	0.9945	0.9945
CALLY	80	−0.122	0.2795	0.4111
CRP	80	0.117	0.3002	0.4169
ESR	80	0.182	0.1064	0.3703
Albumin	80	0.042	0.7136	0.7756
Lymphocyte	80	−0.061	0.5939	0.7070
Neutrophil	80	0.205	0.0680	0.3204
Hemoglobin	80	0.131	0.2464	0.4111
Platelet	80	0.042	0.7127	0.7756
Glucose	80	0.146	0.1956	0.4075
ALT	80	0.132	0.2437	0.4111
AST	80	0.098	0.3881	0.5106
BUN	80	0.170	0.1313	0.3703
Creatinine	80	0.312	0.0048	0.0601
LDL	80	0.150	0.1852	0.4075
HDL	80	−0.169	0.1333	0.3703
Triglycerides	80	0.199	0.0769	0.3204
Cholesterol	80	0.127	0.2633	0.4111
HbA1c	80	0.206	0.0668	0.3204

Spearman’s rank correlation. n = non-missing pairs.

**Table A2:** AS—Spearman correlations of AGE-SAF (FDR-adjusted).

Parameter	n	Spearman r	*p* (Unadjusted)	*q* (BH-FDR)
Disease duration	83	−0.070	0.5323	0.9756
Steroid dose	83	−0.023	0.8337	0.9856
BASDAI	83	0.360	<0.001	0.0194
SII	83	0.079	0.4800	0.9756
HALP	83	−0.043	0.7015	0.9856
CALLY	83	−0.084	0.4510	0.9756
CRP	83	0.000	0.9967	0.9967
ESR	83	0.095	0.3920	0.9756
Albumin	83	−0.124	0.2625	0.9756
Lymphocyte	83	−0.003	0.9752	0.9967
Neutrophil	83	0.153	0.1669	0.9599
Hemoglobin	83	0.001	0.9899	0.9967
Platelet	83	−0.020	0.8571	0.9856
Glucose	83	0.094	0.3970	0.9756
ALT	83	−0.073	0.5113	0.9756
AST	83	−0.032	0.7746	0.9856
BUN	83	−0.038	0.7360	0.9856
Creatinine	83	−0.066	0.5514	0.9756
LDL	83	−0.111	0.3168	0.9756
HDL	83	−0.034	0.7569	0.9856
Triglycerides	83	0.186	0.0917	0.7028
Cholesterol	83	0.034	0.7622	0.9856
HbA1c	83	0.198	0.0723	0.7028

Spearman’s rank correlation. n = non-missing pairs.

**Table A3:** RA—Subgroup comparisons of AGE-SAF (FDR-adjusted).

Comparison (Yes vs. No)	n (Yes)	n (No)	*p* (Unadjusted)	*q* (BH-FDR)
Active disease	35	45	0.0110	0.0219
Comorbidity	61	19	0.1374	0.1717
Diabetes mellitus	12	68	0.0567	0.0945
Coronary artery disease	15	65	0.0039	0.0116
Hypertension	45	35	0.0681	0.0973
Hyperlipidemia	18	62	0.0030	0.0116
Osteoporosis	19	61	0.2293	0.2548
Atherosclerosis	14	66	<0.001	0.0088
Cerebrovascular disease *	6	74	0.0046	0.0116
Major organ involvement *	6	74	0.9854	0.9854

Mann–Whitney U test. Green shading indicates *q* < 0.05. * = subgroup with n < 10; interpret with caution.

**Table A4:** AS—Subgroup comparisons of AGE-SAF (FDR-adjusted).

Comparison (Yes vs. No)	n (Yes)	n (No)	*p* (Unadjusted)	*q* (BH-FDR)
Active disease	37	46	0.0032	0.0146
Comorbidity	40	43	0.0159	0.0437
Diabetes mellitus *	4	79	0.0394	0.0487
Coronary artery disease *	9	74	0.0194	0.0437
Hypertension	19	64	0.0392	0.0487
Hyperlipidemia *	7	76	0.0293	0.0487
Osteoporosis *	3	80	0.0433	0.0487
Atherosclerosis *	7	76	<0.001	0.0026
Cerebrovascular disease *	2	81	0.1247	0.1247

Mann–Whitney U test. Green shading indicates *q* < 0.05. * = subgroup with n < 10; interpret with caution. Note: hypertension, diabetes mellitus, and osteoporosis pass the FDR threshold but are based on very small “yes” subgroups (n ≤ 19) and should be interpreted with extreme caution.
